# Ophthalmic Genetics: Moving Forward

**DOI:** 10.4103/0974-9233.75875

**Published:** 2011

**Authors:** Khaled K. Abu-Amero

**Affiliations:** Department of Ophthalmology, Ophthalmic Genetics Laboratory, College of Medicine, King Saud University, P. O. Box 245, Riyadh 11411, Saudi Arabia

The role of heredity in various ophthalmic diseases has been explored for more than 100 years, with the early focus on conditions that could be identified specifically as straightforward Mendelian inherited disorders. In these disorders, replication of the trait is easily observed and clear-cut probabilities of transmission can be readily calculated. The prevalence of consanguinity within Middle Eastern and African populations is relatively high and this is associated with increased frequency of inherited ocular diseases in this part of the world.^1^ Therefore, the region should be at the forefront in attempting to benefit from recent advances in molecular genetics techniques and in putting these techniques to work for genetic testing and counseling.

Our understanding of the genetics of eye diseases has benefited tremendously from advances in molecular genetics techniques.^2^ This became particularly apparent after the completion of the human genome mapping project, which facilitated the identification of an ever increasing number of human genes. Recently, the advent of exome sequencing (also known as targeted exome capture) to selectively sequence the coding regions of the human genome has certainly increased the likelihood of successfully identifying genes associated with specific familial ocular conditions.^3^ This, in turn, will help in the development of suitable treatments and will improve our ability to decrease disease risk and to slow disease progression.

Unfortunately, the benefit of these advancements remains limited in Middle East and African developing countries.^4^ This is clearly evident through the lack of genomic companies, the limited availability of genetic testing, and the limited number of significant scientific publications coming from this region in comparison with the developed world. Health authorities in the region served by the MEAJO, even though crippled by limited health budgets and concentrating efforts and resources on fundamental heath problems, need to make preventing genetic disorders (including hereditary ophthalmologic diseases) a priority. Certainly it is a challenging, but feasible task.

The purpose of this issue is to provide an up-to-date overview of the current status and future directions of the genetics of various common ophthalmic diseases. Reviews on the genetics and genomics of Keratoconus,^5^ primary congenital glaucoma,^6^ pseudoexfoliation syndrome/glaucoma, Leber hereditary optic neuropathy^7^ and a comprehensive review on the genetic diagnostic methods for inherited eye diseases^8^ are included in this issue.

We are very grateful to our colleagues who have contributed reviews to this special issue of MEAJO and who have served as reviewers for those manuscripts. We hope that this excellent collection of articles will provide the ophthalmologic community with up-to-date knowledge regarding this rapidly growing and changing field of ophthalmic genetics.
